# Books and Authors

**Published:** 1910-10

**Authors:** 


### BOOKS AND AUTHORS

**Manual of Midwifery.** By **Henry Jellett,** B.A., M.D. (Dub. Univ.), F.R.C.P.I., L.M., King's Professor of Midwifery in the School of Physic, Trinity College, Dublin. Second edition. Octavo, pp. 1222. Illustrated. New York: William Wood & Co. 1910. (Cloth, $6.00.)

The material in this work has been prepared by a master, one of large experience too, and he has presented in this edition a treatise as nearly complete as it is possible to make a single volume on so vast a subject. The text contains scarcely a superfluous word, so concisely is it written, and yet it is so perfect in expression that no ambiguity remains in the trail of its compact sentences. The make-up of the book is such that an enormous amount of material is offered in its 1210 pages; the illustrations in plates and drawings are of an artistic type, truthful to nature, depicting obstetric medicine and surgery in a clearly instructive manner, the whole contributing to the creation of one of the most useful of our modern works on obstetrics.

The present edition has been improved in every respect in order to conform to the most advanced ideas and facts developed in recent years. Obsolete material has been eliminated, illustrations have been renewed or changed to meet the last requirements of the science. Scarcely a chapter but that reflects the handiwork of the conscientious teacher in revising his work, in order that it may reflect the science and art of present-day midwifery. A single example of Jellett's progressiveness we may instance. He divides Cesarean section into two forms, the conservative and the radical. In the former the incision in the uterus is sutured after removal of the fetus, the uterus being allowed to remain. In radical Cesarean section, the removal of the fetus is followed by either partial or complete removal of the uterus. The term conservative Cesarean section is applied to the classic operation, and the term radical Cesarean section is made to designate the type generally known as Porro's operation.

All through the book are evidences of the progressive spirit, and we are of the opinion that every one who assumes the role of teacher of obstetrics should possess himself of this book, and proceed to familiarise himself with its doctrines. This does not mean that those who simply practise obstetrics do not need the treatise. Far from it! But teachers of all others cannot afford to do without it.

---

**Disorders of Metabolism and Nutrition.** By **Dr. Carl von Noorden,** Professor of the First Medical Clinic, Vienna. Parts VIII and IX. Inanition and Fattening Cures; Technic of Reduction Cures and Gout. 12 mos, pp. 103 and 112. New York: E. B. Treat & Co. 1910. (Cloth, $1.50 each.)

Part eight of this series, dealing with inanition and fattening cures, and part nine dealing with the technic of reduction cures and gout, comprise four lectures delivered by the author in a post-graduate course for Vienna physicians, in May, 1908. The phenomena of under nutrition and the treatment of obesity to a certain extent go hand in hand. In the former condition, the caloric intake should be increased, whereas in the latter class it should be reduced. The author is an expert in handling the disorders of metabolism and nutrition, and in those two little volumes presents a vast amount of information of value on the two phases of this important subject to which the books are devoted.

---

**Transactions of the fifteenth annual meeting of the American Laryngological, Rhinological, and Otological Society,** held at Atlantic City, N. J., June 3-5, 1909. **Thomas J. Harris,** M.D., Secretary, New York,

This association though comparatively young, publishes a volume of transactions that, in material and general appearance, will compare favorably with any book of society work that is issued. The present volume is a "fat" one without padding, there being no spasmodic attempt by the association to publish a big book merely for the looks of the thing. It is full of excellent material and most of the papers have been discussed with vigor and intelligence. The book is well edited, but we wish the use of diphthongs was dispensed with, and that the pages were made to contain running heads showing the title of the paper below in each instance. This would facilitate reference and improve the appearance of the book.

---

**A Manual of Personal Hygiene:** Proper Living upon a Physiologic Basis. By eminent specialists. Edited by **Walter L. Pyle,** M.D., Assistant Surgeon to the Wills Eye Hospital. Philadelphia. Fourth edition. 12mo, of 472 pages, illustrated. Philadelphia and London: W. B. Saunders Company. 1910. Cloth, $1.50 net.)

It is about ten years since the first edition of this book appeared during which time three revised editions and several reprintings have been sent out. This fourth edition has been revised and changes made deemed of importance. The topic is dealt with under several separate heads, the first relating to the digestive apparatus; the second, to the skin and its appendages; the third, to the vocal and respiratory apparatus; the fourth, to the ear; the fifth, to the eye; the sixth, to the brain and nervous system; the seventh, to physical exercise; the eighth, to body posture; and the ninth, to domestic hygiene. An appendix follows in which miscellaneous matters are presented, not belonging to either of the classified topics. The first section is written by Professor Charles G. Stockton, of Buffalo, whose skill in digestive disorders is world-wide. We have commented on this and other sections of this book when presenting notices of former editions, hence at this time shall content ourselves with remarking, that it would be well if every one who has reached the thinking age, could be made familiar with its teachings. Every school teacher should obtain it and promulgate its doctrines on all suitable occasions. If a copy were in every household for reference and study, less occasion would arise for the visits of the family doctor. We regard it as one of the most useful books in our library.

---

**The Conquest of Disease through Animal Experimentation.** By **James Peter Warbasse,** Surgeon to the German Hospital, Biooklyn. 12 mo, pp. 190. New York and London: D. Appleton & Co. 1910. (Cloth, $1.00.)

This essay, written in ten sections, is based as the author states on addresses delivered before the New York Academy of Medicine, the Medical Society of the County of Kings, and the Brooklyn Institute of Arts and Science. These have been amplified and published in book form, the object being to aid in correcting some misconceptions, and to elucidate an important field of scientific effort. It constitutes an able defense of vivisection; or, perhaps we should say, a masterful attack upon the antivivisection fad, now being carried on by misguided men and women who either do not understand; or knowing, wilfully misrepresent. The subject is presented in a popular manner and should be read by every one interested in the conquest of disease.

---

**Borderland Surgery.** By **Gustavus M. Blech,** M.D., Professor of Clinical Surgery, Medical Department Loyola University, Chicago. 12 mo, pp. 219. Philadelphia: Professional Publishing Co. 1910. (Cloth, $1.50.)

The publishers of this small but useful book have exercised the bad privilege of attempting to review this book under the head of "Publisher's Note," printed immediately after the table of contents. The author in bad taste in his foreword refers to a distinguished surgeon now deceased, as "the late Senn." He also announces himself as professor of clinical surgery in the medical department of Loyola University. Nevertheless, even if so overweighted in its "front," it is a book full of useful hints and suggestions which the beginner in surgical practice may make available when in doubt. It may be of service, too, as a reminder to the older practitioner but it is absurd to talk about carrying it in the pocket.

---

**Index of Symptoms with Diagnostic Methods.** By **Ralph Winnington Leftwich,** M.D., Late Assistant Physician to the East London Children's Hospital. Fourth edition. 12mo, pp. 451. New York: William Wood & Co. 1910. (Cloth, $2.25.)

This small book has proved its popularity by the number of editions that have been required to meet the calls upon the publishers from copies of the work. The author has made a striking success of the index because, as we think, of its practicability and its novelty. So far as we know nothing like it has been attempted before. Its completeness is remarkable and its accuracy is very nearly absolute. The section on diagnosis in the introduction, especially the classification of patients and the "fallacies" pointed out, are instructive, as are, also, the diagnostic paragraphs at the head of many of the sections. It will repay any physician to keep this index at hand, it being both a reminder and a timesaver.

---

**The Pathology of the Living and Other Essays.** By **B. G. A. Moynihan,** M.S. (London), F.R.C.S., Honorary Surgeon to Leeds General Infirmary; Professor of Clinical Surgery at the University of Leeds, England. 12mo, of 260 pages. Philadelphia and London: W. B. Saunders Company, 1910. (Cloth, $2.00 net.)

A number of essays that have appeared from time to time in various medical journals, written by Mr. Moynihan, have been gathered into this volume, and they make exceedingly interesting reading. Mr. Moynihan is an observing surgeon as well as a skilful operator, and he is contending for a reduction of the so-called "functional diseases," most of which are dependent upon demonstrable changes in structure. Through his own investigations and those of others along the same or similar lines, most of the conditions for a long time grouped as dyspepsias, have been turned over to the surgeon of late, and he has found them to be stomach or duodenal ulcer, some one of the various forms of cholelithiasis, or chronic appendicitis. In his remarks on the early diagnosis and treatment of cancer of the stomach, the author makes the startling statement that this disease claims about 1,500 victims every year in England. But this group of instructive essays should be read to be appreciated. They are written in a style that entertains and teaches at one and the same time, and they should be read by every physician who attempts the treatment of dyspepsia.

---

**The Medical Epitome Series.** Diseases of the Skin. A Manual for Students and Practitioners. By **Alfred Schalek,** M.D., Professor of Dermatology, University of Nebraska. Second edition. 12 mo, 255 pages, with 47 engravings. Lea & Febiger, Publishers, Philadelphia and New York, 1910. (Cloth ,$1.00, net.)

An epitome such as this is of use frequently to one who wishes to reach the gist of some topic in haste. The material is well prepared for the purpose named and the illustrations are excellent.

